# Arsenite Inhibits Tissue-Type Plasminogen Activator Synthesis through NRF2 Activation in Cultured Human Vascular Endothelial EA.hy926 Cells

**DOI:** 10.3390/ijms22020739

**Published:** 2021-01-13

**Authors:** Tsuyoshi Nakano, Tsutomu Takahashi, Chika Yamamoto, Eiko Yoshida, Toshiyuki Kaji, Yasuyuki Fujiwara

**Affiliations:** 1 Department of Environmental Health, School of Pharmacy, Tokyo University of Pharmacy and Life Sciences, 1432-1 Horinouchi, Hachioji 192-0392, Japan; y111125@toyaku.ac.jp (T.N.); tsutomu@toyaku.ac.jp (T.T.); 2 Department of Environmental Health, Faculty of Pharmaceutical Sciences, Toho University, 2-2-1 Miyama, Funabashi 274-8510, Japan; yamamoto@phar.toho-u.ac.jp; 3 Department of Environmental Health, Faculty of Pharmaceutical Sciences, Tokyo University of Science, 2641 Yamazaki, Noda 278-8510, Japan; eyoshida@rs.tus.ac.jp

**Keywords:** arsenite, nuclear factor erythroid 2 related factor 2, tissue-type plasminogen activator, fibrinolysis, endothelial cell, atherosclerosis

## Abstract

Chronic arsenic exposure is known to be related to the progression of atherosclerosis. However, the pathogenic mechanisms of arsenic-induced atherosclerosis have not been fully elucidated. Because disruption of the blood coagulation/fibrinolytic system is involved in the development of arteriosclerosis, we investigated the effect of arsenite on fibrinolytic activity in human vascular endothelial EA.hy926 cells in the present study. Fibrinolysis depends on the balance between tissue-type plasminogen activator (t-PA) and plasminogen activator inhibitor 1 (PAI-1) secreted from vascular endothelial cells. We found that arsenite reduced fibrinolytic t-PA activity by inhibiting its synthesis without affecting PAI-1 production. The inhibitory effect of arsenite on t-PA expression was partially recovered by the reactive oxygen species (ROS) scavenger Trolox. The nuclear factor erythroid 2 related factor 2 (NRF2) pathway is known to be activated by arsenite via ROS production. We confirmed that arsenite activated the NRF2 pathway, and arsenite-induced inhibition of fibrinolytic t-PA activity was abrogated in NRF2-knockdown EA.hy926 cells. These results suggest that arsenite inhibits the fibrinolytic activity of t-PA by selectively suppressing its synthesis via activation of the NRF2 pathway in vascular endothelial cells.

## 1. Introduction

Arsenic is a ubiquitous metalloid found in the Earth’s crust. Arsenic pollution of groundwater has been reported in several countries across the globe [1,2,3]. Recently, Podgorski and Berg estimated that 94 to 220 million people are potentially exposed to high concentrations of arsenic in groundwater worldwide [3]. Chronic arsenic exposure has been implicated in the development of various diseases, such as skin lesions, hypertension, cardiovascular disease, peripheral vascular disorders (e.g., Blackfoot disease), neuropathy, cancer, and arteriosclerosis [4,5,6,7,8,9,10,11]. Therefore, elucidating the mechanism of arsenic toxicity is important for the prevention and treatment of these diseases.

In animal experiments using apolipoprotein E-deficient mice as a model of human atherosclerosis, it has also been shown that exposure to arsenite accelerates the progression of atherosclerosis [12,13,14,15]. Although the pathogenic mechanism of atherosclerosis is particularly complicated, the disease is generally initiated by functional damage to vascular endothelial cells, followed by monocyte/macrophage invasion into the subendothelium tissue. As a result, vascular smooth muscle cells are transformed from a contractile phenotype to a synthetic phenotype and actively proliferate to induce intimal hyperplasia in the vascular wall [16]. In addition, perturbation of the blood coagulation/fibrinolytic system in vascular tissue is known to be involved in the development of atherosclerosis [17,18,19]. Normally, blood flow is maintained without clotting or excessive bleeding by the delicate balance between blood coagulation and fibrinolysis [20]. In particular, plasmin converted from plasminogen by tissue-type plasminogen activator (t-PA) secreted from endothelial cells is responsible for this fibrinolytic activity [21,22]. Because t-PA activity is inhibited by plasminogen activator inhibitor 1 (PAI-1) secreted from endothelial cells [23,24,25], endothelial fibrinolytic activity depends on the balance between t-PA and PAI-1 produced by vascular endothelial cells. Previous reports have shown that patients with Blackfoot disease exhibit disorders of the fibrinolytic system [26]. Arsenite decreases fibrinolytic activity in human microvascular endothelial cells (HMECs), but not human umbilical vein endothelial cells (HUVECs), via both a reduction in t-PA expression and an increase in PAI-1 expression [27]. It is well known that the cyclic adenosine monophosphate (AMP) pathway negatively regulates t-PA synthesis, and the protein kinase C pathway positively mediates PAI-1 synthesis [28,29]. However, the detailed mechanism underlying the inhibition of fibrinolytic activity in vascular endothelial cells by arsenite remains largely unknown.

The human-derived endothelial cell line EA.hy926 was established by hybridization [30]. Cui et al. [31] reported that low concentrations of arsenite stimulate cell migration and tube formation in endothelial EA.hy926 cells and HMECs [32]. Recently, we have shown that the transcription factor nuclear factor erythroid 2 related factor 2 (NRF2) is a negative regulator of t-PA synthesis in endothelial EA.hy926 cells [33]. To elucidate the pathogenic mechanisms of peripheral vascular disorders and atherosclerotic disease caused by arsenic exposure in this study, we investigated the effects of arsenite on the fibrinolytic system in vascular endothelial cells and its detailed mechanisms using a culture system of endothelial EA.hy926 cells. Here, we report that arsenite inhibits fibrinolytic activity through NRF2 pathway activation in EA.hy926 cells.

## 2. Results

### 2.1. Arsenite Inhibits Fibrinolytic Activity in Endothelial EA.hy926 Cells without Inducing Nonspecific Cell Damage

**Figure 1:**
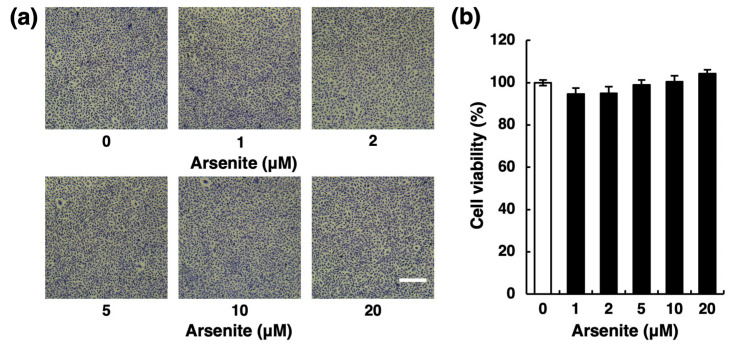
Effect of arsenite on the cytotoxicity of human vascular endothelial EA.hy926 cells. (**a**) Morphological appearance of endothelial EA.hy926 cells after exposure to arsenite at 1, 2, 5, 10, or 20 µM for 24 h. Scale bar = 400 µm. (**b**) The cell viability of endothelial EA.hy926 cells after exposure to arsenite at 1, 2, 5, 10, or 20 µM for 24 h. The data are reported as the mean ± S.D. of four samples. The data were analyzed using one-way ANOVA, followed by the Bonferroni/Dunn test.

We first confirmed the cytotoxicity of arsenite (NaAsO_2_) against endothelial EA.hy926 cells by morphological observation and 3-(4,5-dimethyl-2-thiazolyl)-2,5-diphenyl-2H-tetrazolium bromide (MTT) cell viability assays. After a 24-h incubation, arsenite did not alter the morphology of cell layers (Figure 1a). In addition, arsenite treatment did not compromise cell viability (Figure 1b), even after exposure for 48 h (Figure S1). These results indicate that arsenite did not cause nonspecific cell damage under these experimental conditions.

**Figure 2:**
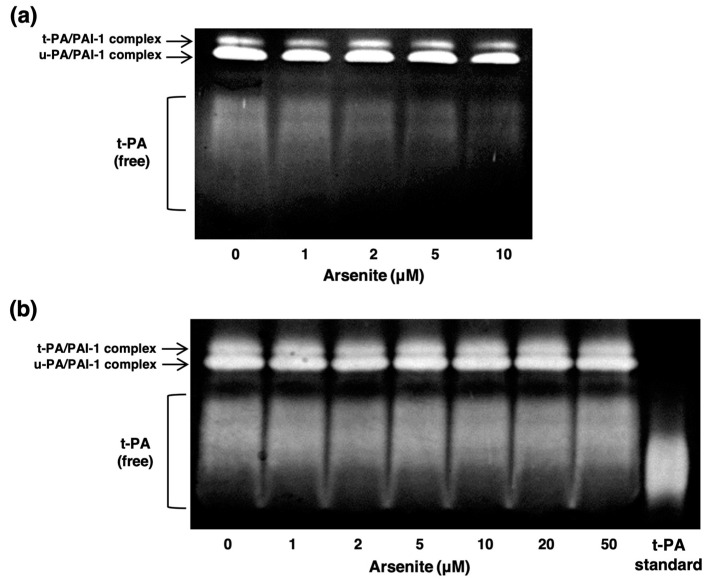
Effect of arsenite on the fibrinolytic activity of endothelial EA.hy926 cells. (**a**) Fibrin zymography of conditioned medium collected from endothelial EA.hy926 cells after exposure to arsenite. The cells were incubated at 37 °C for 24 h with arsenite at 1, 2, 5, or 10 µM. (**b**) Fibrin zymography of conditioned medium collected from endothelial EA.hy926 cells incubated with arsenite under cell-free conditions. The cells were incubated at 37 °C for 24 h in the absence of arsenite, and then the conditioned medium was incubated at 37 °C for 24 h in the presence of arsenite at 1, 2, 5, 10, 20, or 50 µM under cell-free conditions.

Next, we examined the effects of arsenite on fibrinolytic activity under nontoxic conditions in endothelial EA.hy926 cells. Figure 2a shows the fibrin zymography of conditioned medium collected from endothelial EA.hy926 cells treated with arsenite for 24 h. Lower lytic zones, which indicate fibrinolytic free t-PA activity, were observed in broad bands, and t-PA activity in the conditioned medium was decreased by arsenite treatment in a concentration-dependent manner. Because arsenite is known to bind to proteins and inhibit their activity [34,35], it is possible that arsenite directly inhibits t-PA activity in the conditioned medium via direct binding to t-PA. However, t-PA activity in the conditioned medium was not affected by incubation with arsenite concentrations up to 50 µM under cell-free conditions, indicating that arsenite inhibited endothelial fibrinolytic activity without directly inhibiting t-PA activity (Figure 2b). In other words, arsenite reduced t-PA activity by inhibiting fibrinolytic protein synthesis, leading to endothelial cell dysfunction.

### 2.2. Arsenite, but Not Arsenate, Selectively Inhibits Endothelial t-PA Synthesis

**Figure 3:**
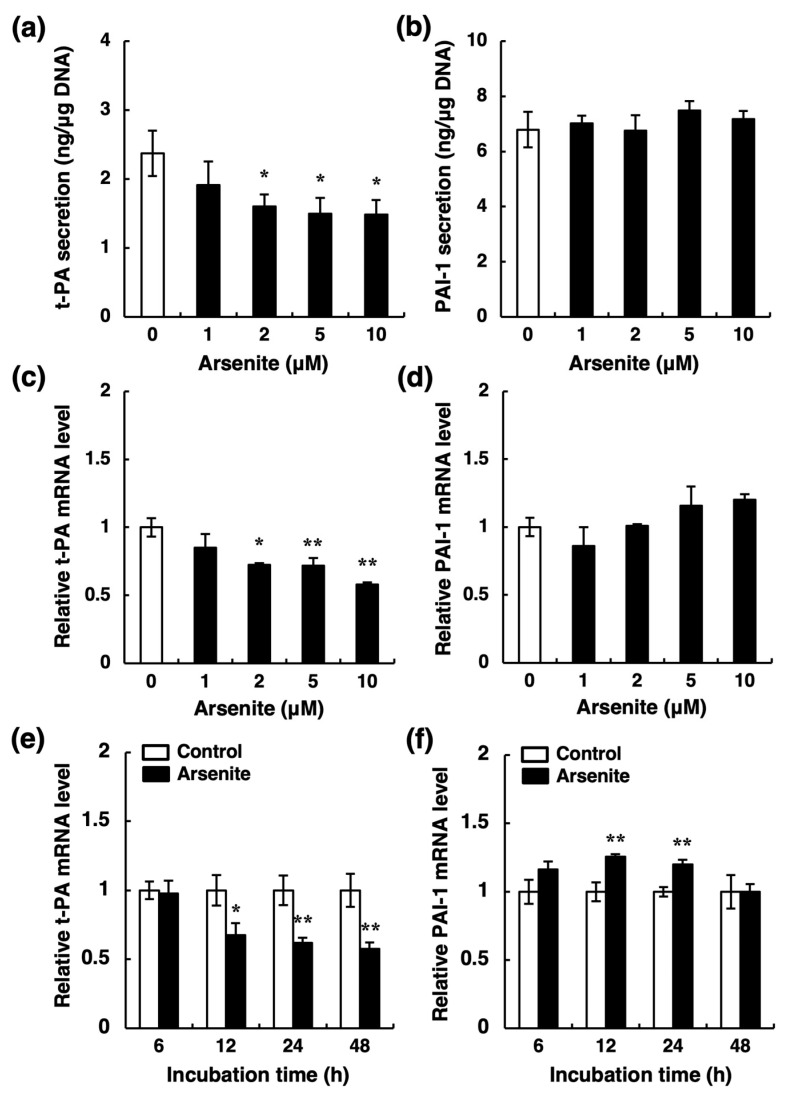
Effects of arsenite on the expression and secretion of fibrinolytic proteins in endothelial EA.hy926 cells. The accumulation of t-PA (**a**) and PAI-1 (**b**) in the conditioned medium of endothelial EA.hy926 cells. The cells were incubated with arsenite at 1, 2, 5, or 10 µM for 24 h. The data are reported as the mean ± S.D. of four samples. The data were analyzed using one-way ANOVA, followed by the Bonferroni/Dunn test. * Significantly different from the control, *p* < 0.05. The mRNA expression of t-PA (**c**) and PAI-1 (**d**) in endothelial EA.hy926 cells. The cells were incubated with arsenite at 1, 2, 5, or 10 µM for 24 h. The data are reported as the mean ± S.D. of three samples. The data were analyzed using one-way ANOVA, followed by the Bonferroni/Dunn test. Significantly different from the control, * *p* < 0.05; ** *p* < 0.01. The mRNA expression of t-PA (**e**) and PAI-1 (**f**) in endothelial EA.hy926 cells. The cells were incubated with arsenite at 10 µM for 6, 12, 24, or 48 h. The data are reported as the mean ± S.D. of three samples. The data were analyzed using Student’s *t*-test. Significantly different from the corresponding control, * *p* < 0.05; ** *p* < 0.01.

**Figure 4:**
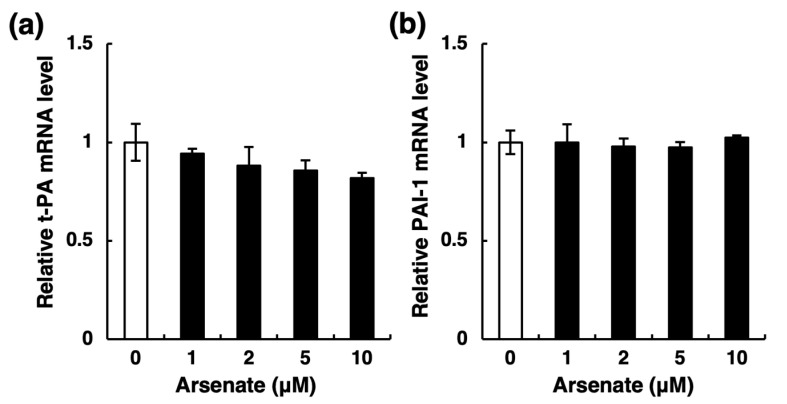
Effects of arsenate on the mRNA expression of t-PA (**a**) and PAI-1 (**b**) in endothelial EA.hy926 cells. The cells were incubated with arsenate at 1, 2, 5, or 10 µM for 24 h. The data are reported as the mean ± S.D. of three samples. The data were analyzed using one-way ANOVA, followed by the Bonferroni/Dunn test.

Fibrinolytic activity depends on the balance between t-PA and PAI-1 [20]. Thus, there are two possibilities regarding the decrease in t-PA activity: a reduction in t-PA synthesis and an increase in PAI-1 synthesis in vascular endothelial cells. To examine these possibilities, we next determined the secretion levels of t-PA and PAI-1 proteins into the conditioned medium of endothelial EA.hy926 cells. After a 24-h treatment, arsenite at ≥2 µM significantly decreased the accumulation of t-PA in the conditioned medium of these cells (Figure 3a) without affecting PAI-1 (Figure 3b). Furthermore, the level of t-PA mRNA was also significantly decreased by arsenite at ≥2 µM (Figure 3c), whereas that of PAI-1 mRNA was unchanged (Figure 3d). These results suggest that the decreased t-PA activity in the conditioned medium of endothelial EA.hy926 cells results from the selective inhibition of t-PA synthesis by arsenite. In addition, the selective inhibition of t-PA expression was observed after a 12-h treatment with arsenite and continued until 48 h (Figure 3e), whereas PAI-1 expression was slightly increased after 12 and 24 h (Figure 3f). In contrast, arsenite treatment did not affect urokinase-type plasminogen activator (u-PA) mRNA expression (Figure S2). Furthermore, arsenate (Na_2_HAsO_4_), a pentavalent arsenic compound, only slightly decreased the mRNA expression of t-PA (Figure 4a); the mRNA expression of PAI-1 was not changed by arsenate (Figure 4b). Therefore, these results indicate that arsenite (As^III^), but not arsenate (As^V^), significantly inhibits the fibrinolytic activity of vascular endothelial cells via the selective suppression of t-PA synthesis and secretion.

### 2.3. Arsenite Inhibits t-PA Synthesis via the NRF2 Pathway

Next, we investigated the molecular pathway involved in the suppression of t-PA synthesis by arsenite in endothelial EA.hy926 cells. It has been reported that the cyclic AMP pathway contributes to the suppression of endothelial t-PA production [28]. However, the release of prostacyclin (PGI_2_), which stimulates the cyclic AMP pathway via activation of adenylate cyclase from endothelial EA.hy926 cells into the conditioned medium, did not change after arsenite treatment (Figure S3a). In addition, arsenite-induced suppression of t-PA expression was not recovered by SQ22536, an adenylate cyclase inhibitor (Figure S3b). These results suggest that the cyclic AMP pathway is not involved in the suppression of t-PA synthesis by arsenite in endothelial EA.hy926 cells.

**Figure 5:**
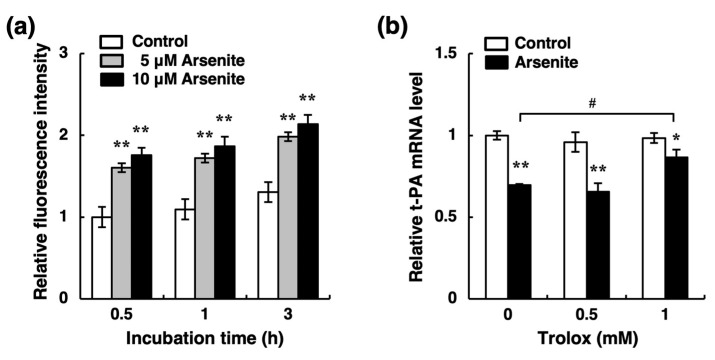
Possible involvement of reactive oxygen species (ROS) production in the inhibition of t-PA mRNA expression by arsenite in endothelial EA.hy926 cells. (**a**) Effect of arsenite on the levels of intracellular ROS in endothelial EA.hy926 cells. The cells were pretreated with 2′,7′-dichlorodihydrofluorescein diacetate (H_2_DCFDA) at 100 µM for 1 h and then treated with arsenite at 5 or 10 µM for 0.5, 1, or 3 h. The data are reported as mean ± S.D. of six samples. The data were analyzed using one-way ANOVA, followed by the Bonferroni/Dunn test. ** Significantly different from the corresponding control, *p* < 0.01. (**b**) Effect of Trolox, a radical scavenger, on arsenite-induced suppression of t-PA mRNA expression in endothelial EA.hy926 cells. The cells were treated with arsenite at 10 µM in the presence of Trolox at 0.5 or 1 mM for 24 h. The data are reported as the mean ± S.D. of three samples. The data were analyzed using one-way ANOVA, followed by the Bonferroni/Dunn test. Significantly different from the corresponding control, * *p* < 0.05; ** *p* < 0.01. ^#^ Significantly different from the arsenite-treated cells without Trolox, *p* < 0.05.

**Figure 6:**
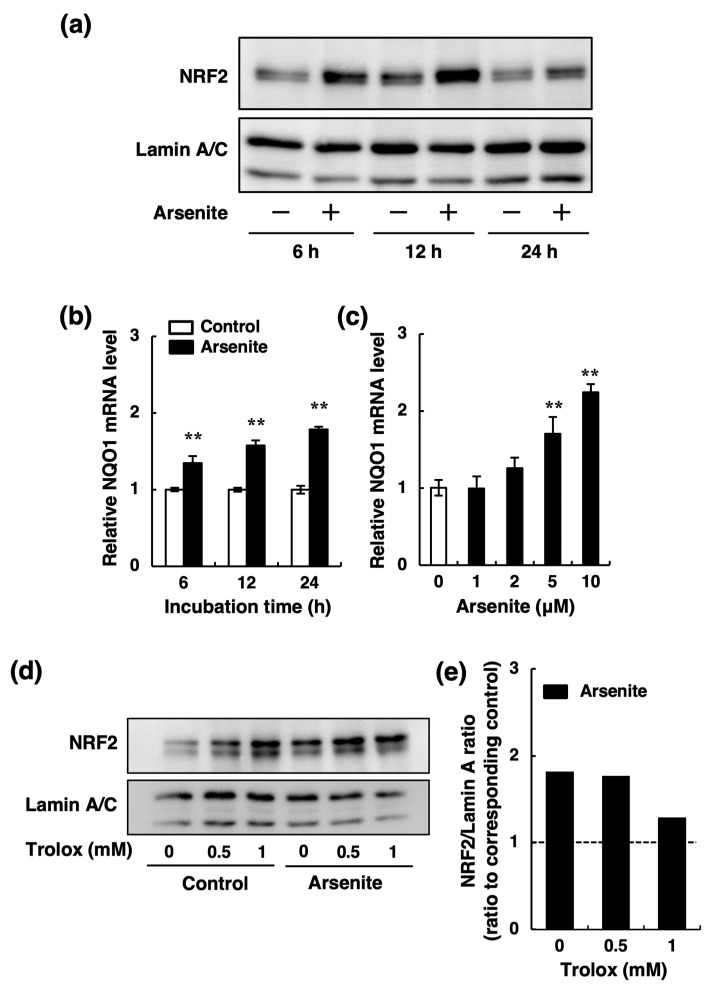
Effect of arsenite on NRF2 pathway activation in endothelial EA.hy926 cells. (**a**) Intranuclear NRF2 levels in endothelial EA.hy926 cells. The cells were incubated with arsenite at 10 µM for 6, 12, or 24 h. (+) indicates treated with arsenite; (−) indicates treated without arsenite. (**b**) The expression levels of NQO1 mRNA in endothelial EA.hy926 cells. The cells were incubated with arsenite at 10 µM for 6, 12, or 24 h. The data are reported as the mean ± S.D. of three samples. The data were analyzed using Student’s *t*-test. ** Significantly different from the corresponding control, *p* < 0.01. (**c**) The expression levels of NQO1 mRNA in endothelial EA.hy926 cells. The cells were incubated with arsenite at 1, 2, 5, or 10 µM for 24 h. The data are reported as the mean ± S.D. of three samples. The data were analyzed using one-way ANOVA, followed by the Bonferroni/Dunn test. ** Significantly different from the control, *p* < 0.01. (**d**) Intranuclear NRF2 levels in endothelial EA.hy926 cells. The cells were incubated with arsenite at 10 µM in the presence of Trolox at 0.5 and 1 mM for 24 h. (**e**) Ratio of NRF2/Lamin A in arsenite-treated groups to that in the corresponding control groups in (**d**).

Recently, we showed that activation of the NRF2 pathway decreases endothelial t-PA synthesis in endothelial EA.hy926 cells [33]. Because it is well known that arsenite activates the NRF2 pathway via increased reactive oxygen species (ROS) production [35,36,37], we examined the possible involvement of ROS production in the inhibition of t-PA mRNA expression by arsenite in endothelial EA.hy926 cells. As shown in Figure 5a, treatment of endothelial EA.hy926 cells with arsenite at 5 and 10 µM significantly increased the levels of intracellular ROS after 0.5, 1, and 3 h. Moreover, the inhibitory effect of arsenite on t-PA expression was partially recovered by Trolox, a natural ROS scavenger (Figure 5b). We next examined whether arsenite activates the NRF2 pathway in endothelial EA.hy926 cells. As shown in Figure 6a, the intranuclear NRF2 protein levels in endothelial EA.hy926 cells were upregulated after 6, 12, and 24 h of treatment with 10 µM arsenite. Similarly, the expression level of NAD(P)H quinone dehydrogenase 1 (NQO1) mRNA, a downstream target of NRF2, was upregulated by arsenite (Figure 6b). In a dose-response experiment, arsenite at ≥5 µM significantly increased the expression of NQO1 mRNA after 24 h of exposure (Figure 6c). To confirm that Trolox inhibits the activation of the NRF2 pathway induced by arsenite, we examined the effect of Trolox on arsenite-induced intranuclear NRF2 protein levels. As shown in Figure 6d, Trolox alone increased the NRF2 protein level, as previously reported [38,39], although it did not change the t-PA mRNA expression (Figure 5b). When we calculated the ratio of NRF2/Lamin A in the arsenite-treated groups to that in the corresponding control groups, and we found that the arsenite-induced increase in the NRF2 protein ratio was decreased by 1-mM Trolox treatment (Figure 6e). In other words, arsenite did not activate NRF2 in the presence of Trolox. These results suggest that arsenite promotes the nuclear translocation of NRF2 and that subsequent activation of the NRF2 pathway and that ROS production induced by arsenite is partly involved in the activation of NRF2 in endothelial EA.hy926 cells. 

**Figure 7:**
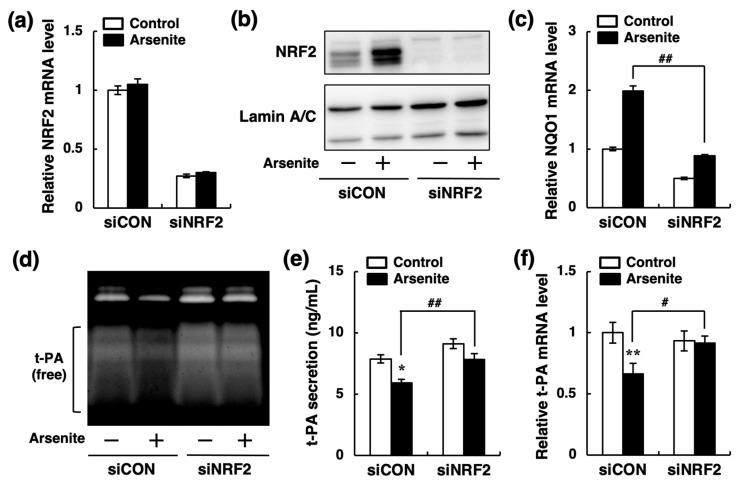
Possible involvement of the NRF2 pathway in the inhibition of t-PA expression by arsenite in endothelial EA.hy926 cells. (**a**) The expression level of NRF2 mRNA in endothelial EA.hy926 cells transfected with control siRNA (siCON) or NRF2 siRNA (siNRF2). The cells were incubated with arsenite at 10 µM for 24 h. The data are reported as the mean ± S.D. of three samples. (**b**) The protein level of NRF2 in siCON or siNRF2 transfected endothelial EA.hy926 cells after exposure to arsenite at 10 µM for 24 h. (+) indicates treated with arsenite; (−) indicates treated without arsenite. (**c**) The expression levels of NQO1 mRNA in siCON and siNRF2 transfected endothelial EA.hy926 cells after exposure to arsenite at 10 µM for 24 h. The data are reported as the mean ± S.D. of three samples. The data were analyzed using one-way ANOVA, followed by the Bonferroni/Dunn test. ^##^ Significantly different from siCON transfected cells treated with arsenite, *p* < 0.01. (**d**) Fibrin zymography of conditioned medium collected from siCON or siNRF2 transfected endothelial EA.hy926 cells after exposure to arsenite at 10 µM for 24 h. (**e**) The accumulation of t-PA in the conditioned medium of siCON or siNRF2 transfected endothelial EA.hy926 cells. The cells were incubated in the presence or absence of arsenite at 10 µM for 24 h. The data are reported as the mean ± S.D. of four samples. The data were analyzed using one-way ANOVA, followed by the Bonferroni/Dunn test. * Significantly different from the corresponding control, *p* < 0.05. ^##^ Significantly different from the corresponding siCON transfected cells, *p* < 0.01. (**f**) The expression level of t-PA mRNA in siCON or siNRF2 transfected endothelial EA.hy926 cells. The cells were incubated with arsenite at 10 µM for 24 h. The data are reported as the mean ± S.D. of three samples. The data were analyzed using one-way ANOVA, followed by the Bonferroni/Dunn test. ** Significantly different from the corresponding control, *p* < 0.01. ^#^ Significantly different from the corresponding siCON transfected cells, *p* < 0.05.

To investigate the involvement of the NRF2 pathway in the suppression of t-PA synthesis by arsenite, we next prepared NRF2-knockdown endothelial EA.hy926 cells by NRF2 siRNA transfection. In NRF2 siRNA transfected cells, both NRF2 mRNA and intranuclear protein levels were significantly decreased; arsenite did not increase the intranuclear NRF2 protein level (Figure 7a,b). In addition, arsenite did not increase NQO1 mRNA in NRF2-knockdown cells (Figure 7c). Moreover, the inhibition of t-PA fibrinolytic activity in the conditioned medium of endothelial EA.hy926 cells by arsenite was abrogated by NRF2 knockdown (Figure 7d). Furthermore, NRF2 knockdown significantly restored both the t-PA protein secretion and t-PA mRNA expression that were reduced by arsenite (Figure 7e,f). These results indicate that arsenite inhibits the fibrinolytic activity of t-PA by suppressing t-PA synthesis via activation of the NRF2 pathway in endothelial EA.hy.926 cells.

## 3. Discussion

**Figure 8:**
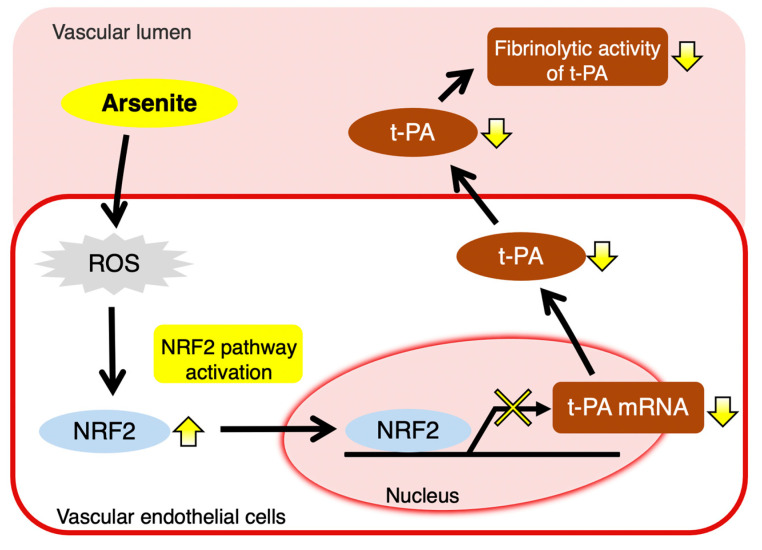
The intracellular signaling pathway that mediates arsenite-induced suppression of t-PA expression in vascular endothelial cells. Arsenite activates NRF2 transcriptional activity partly by enhancing ROS production. Activation of the NRF2 pathway reduces t-PA expression and secretion, resulting in a decrease in the fibrinolytic activity of t-PA. In this study, the mechanism underlying the inhibition of fibrinolysis by arsenite in human vascular endothelial cells was clarified.

In the present study, we found that arsenite (As^III^) inhibits t-PA synthesis through NRF2 activation in cultured human vascular endothelial EA.hy926 cells without altering PAI-1 synthesis, resulting in decreased fibrinolytic activity (Figure 8). To our knowledge, this is the first report to elucidate a part of the molecular mechanism underlying the inhibition of fibrinolytic t-PA activity in vascular endothelial cells exposed to arsenite. Previously, Jiang et al. showed that treatment with arsenite resulted in the inhibition of t-PA synthesis and stimulation of PAI-1 synthesis in HMECs, but not HUVECs [27]. It is suggested that the mechanism contributing to the decreased fibrinolytic activity induced by arsenic may differ depending on the type of vascular endothelial cells. We also demonstrated that arsenite activated the NRF2 pathway partially through increasing ROS production in endothelial EA.hy926 cells. Arsenite is known to rapidly induce ROS formation through activation of the NADPH oxidase (Nox) isoform Nox2 in vascular endothelial cells [35,40]. Thus, Nox2 may also be activated by arsenite in endothelial EA.hy926 cells. However, whether arsenite activates Nox2 to induce ROS production in endothelial EA.hy926 cells requires further study. 

Previous reports have shown that arsenic activates the NRF2 pathway, leading to the upregulation of antioxidant proteins (such as heme oxygenase-I), phase II proteins (such as glutathione *S*-transferases), and phase-III transporters (such as multidrug resistance-associated proteins) [36,37,41]. Additionally, Shinkai et al. reported that activation of NRF2 by the NRF2 activator sulforaphane upregulates these proteins and diminishes both arsenite toxicity and arsenic accumulation in primary mouse hepatocytes [42]. These reports clearly indicate that NRF2 activation is a protective response against arsenite toxicity. However, NRF2 activation is also involved in the development of vascular diseases, such as atherosclerosis, by decreasing the fibrinolytic activity of t-PA in vascular endothelial cells, as shown in this study. It has also been reported that the disruption of NRF2 expression attenuates the development of atherosclerosis in apolipoprotein E-deficient mice [43,44]. This result shows that NRF2 is a modifier of atherosclerosis and that it exhibits pro-atherogenic functions. Furthermore, Sussan et al. [43] showed that the pro-atherogenic effect of NRF2 is mediated via the positive regulation of CD36, which is associated with modified low-density lipoprotein uptake and foam cell formation in macrophages. NRF2 activation is also involved in the regulation of macrophage polarization [45]. We are currently studying the effect of arsenite on the expression of coagulation- and fibrinolysis-related factors in macrophages.

In addition to arsenic, the toxic metals lead and cadmium have been shown to target the vascular system [46]. We previously reported that lead and cadmium, which can cause vascular disease [47,48], decreased fibrinolytic activity in HUVECs through inhibition of t-PA secretion and stimulation of PAI-1 production, respectively [49,50]. These observations suggest that toxic metals, including arsenic, inhibit the fibrinolytic activity of vascular endothelial cells through different mechanisms and may increase the risk of intravascular blood coagulation and subsequent thrombotic vascular lesions, including atherosclerosis. In addition, given that lead can induce NRF2 pathway activation in vascular endothelial cells [51], it is possible that lead inhibits t-PA synthesis [49] via NRF2 pathway activation, similar to arsenite. Further studies on the perturbation of endothelial cell fibrinolytic activity by toxic metal(oid)s, including lead and cadmium, should be performed to clarify the molecular mechanisms, including the intracellular signaling pathways that mediate the toxicity of these substances.

Further, vascular endothelial cells synthesize heparan sulfate proteoglycans, such as perlecan, and chondroitin/dermatan sulfate proteoglycans, such as biglycan [52,53]. Perlecan and biglycan exhibit anti-thrombin activity through the activation of antithrombin III and heparin cofactor II, respectively [54,55]. We previously showed that arsenite inhibits the expression of both perlecan and biglycan in vascular endothelial cells [56,57]. Thus, exposure to arsenite is thought to promote blood coagulation by inducing vascular endothelial cell dysfunction, including inhibition of the production of both t-PA and proteoglycans [56,57].

In conclusion, we propose a novel mechanism involved in the development of vascular disease induced by arsenic exposure. Specifically, we revealed that arsenic selectively inhibits t-PA synthesis via activation of the transcription factor NRF2 in vascular endothelial cells, resulting in decreased fibrinolytic activity. Although more detailed mechanisms contributing to the arsenite-induced inhibition of t-PA synthesis via the NRF2 pathway are not yet clear, inhibition of the fibrinolytic activity of t-PA by arsenite may be related to the progression of cardiovascular disease, including atherosclerosis, and microvascular disease.

## 4. Materials and Methods 

### 4.1. Cell Culture

The human endothelial cell line EA.hy926 (ATTC, Manassas, VA, USA) [30] was cultured in Dulbecco’s modified Eagle’s medium (DMEM, Fujifilm Wako Pure Chemical Co., Ltd., Osaka, Japan) supplemented with 10% heat-inactivated fetal bovine serum (FBS, Biowest, Nuaillé, France) (10% FBS-DMEM) in a humidified 5% CO_2_ atmosphere at 37 °C. When each experiment was performed, EA.hy926 cells grown in 100-mm dishes were transferred to 24-well culture plates or 6-well plates at a density of 4 × 10^4^ cells/cm^2^ and cultured until confluent.

### 4.2. Morphological Observation and Cell Viability Assay

Endothelial EA.hy926 cells were transferred to 24-well culture plates and cultured until confluent. The cells were treated with arsenite (NaAsO_2_, Fujifilm Wako Pure Chemical Co., Ltd.) at 1, 2, 5, 10, or 20 µM and incubated at 37 °C for 24 or 48 h. After treatment, the medium was discarded, and the cells were washed twice with Dulbecco’s phosphate-buffered saline (D-PBS, Fujifilm Wako Pure Chemical Co., Ltd.). The cells were fixed with methanol and stained with Giemsa solution (Merck KGaA, Darmstadt, Germany). The cell layer was observed morphologically using a DMi1 inverted microscope (Leica Microsystem, Wetzlar, Germany). Separately, cell viability was measured using MTT (Dojindo Laboratories, Kumamoto, Japan). Briefly, after treatment with arsenite, the culture medium was changed to fresh 10% FBS-DMEM containing 0.5 mg/ml MTT, and cells were incubated for 4 h at 37 °C. After removing the medium, dimethyl sulfoxide (Fujifilm Wako Pure Chemical Co., Ltd.) was added to MTT formazan. Absorbance at 570 nm was measured by a Multiskan FC microplate reader (Thermo Fisher Scientific, Waltham, MA, USA).

### 4.3. Fibrin Zymography

Fibrin zymography was performed as described previously with minor modifications [33]. The conditioned medium from endothelial EA.hy926 cells treated with 1, 2, 5, or 10 µM arsenite for 24 h at 37 °C in 24-well culture plates was used to measure fibrinolytic activity. Sodium dodecyl sulfate (SDS)-polyacrylamide gel electrophoresis was performed using a 7.5% running gel and 4.5% stacking gel. The running gel containing fibrin was prepared using plasminogen-rich fibrinogen (0.5 mg/mL) from bovine plasma (Sigma-Aldrich, St. Louis, MO, USA) and thrombin (10 National Institutes of Health unit) from human plasma (Sigma-Aldrich). The conditioned medium was incubated with a 0.15 M Tris-HCl buffer solution containing 3% SDS, 30% glycerol, and 0.03% bromophenol blue at 37 °C for 1 h under a non-reducing condition. After SDS-polyacrylamide gel electrophoresis, the gel was washed twice with 2.5% Triton X-100 for 30 min and incubated with 0.1 M glycine-NaOH buffer (pH 8.3) for 24 h at 37 °C. Then, the gel was stained with 9% acetic acid, 55% methanol, and 0.25% Coomassie brilliant blue solution for 1 h and de-stained with 7.5% acetic acid and 5% methanol to detect the lytic zones indicating the fibrinolytic activity of t-PA. Separately, the conditioned medium of endothelial EA.hy926 cells cultured in the absence of arsenite for 24 h at 37 °C was incubated with 1, 2, 5, 10, 20, or 50 µM arsenite for 24 h at 37 °C under cell-free conditions. After incubation, fibrin zymography of the medium was performed as mentioned above.

### 4.4. Measurement of t-PA, PAI-1, and PGI_2_ Secretion 

The conditioned medium of endothelial EA.hy926 cells treated with 1, 2, 5, or 10 µM arsenite for 24 h at 37 °C in a 24-well culture plate was used to measure t-PA and PAI-1 secretion with an enzyme-linked immunosorbent assay kit (AssayPro LLC, St. Charles, MO, USA), after which the cell layer was analyzed for DNA content using a fluorometric method [58] to normalize the t-PA and PAI-1 content in the conditioned medium per µg DNA. Separately, the conditioned medium of endothelial EA.hy926 cells treated with arsenite for 24 h was used to assess PGI_2_ secretion measured as 6-keto PGF_1α_ with a 6-keto PGF_1α_ enzyme-linked immunosorbent assay kit (Cayman Chemical Co., Ann Arbor, MI, USA). The accumulated 6-keto PGF_1α_ in the conditioned medium was expressed as pg/µg DNA.

### 4.5. siRNA Transfection

Double-strand control small interfering RNA (siRNA) and NRF2 siRNA (CAAACUGACAGAAGUUGACAAUUAU) were purchased from Sigma-Aldrich and Thermo Fisher Scientific, respectively. EA.hy926 cells were transfected with siRNA using Lipofectamine RNAiMAX transfection reagent (Thermo Fisher Scientific) in accordance with the manufacturer’s protocol. Briefly, a double-strand siRNA solution (10 pmol: final siRNA used per well) was added to RNAiMAX transfection reagent and incubated for 10 min at room temperature to generate siRNA/RNAiMAX complexes. After incubation, the complexes were added to endothelial EA.hy926 cells, and the cells were cultured until confluence before each experiment.

### 4.6. Total RNA Isolation

Endothelial EA.hy926 cells were treated with arsenite at 1, 2, 5, 10, or 20 µM or arsenate (Na_2_HAsO_4_, Fujifilm Wako Pure Chemical Co., Ltd.) at 1, 2, 5, or 10 µM in the presence or absence of the radical scavenger Trolox (Fujifilm Wako Pure Chemical Co., Ltd.) at 0.5 or 1 mM for 6, 12, 24, or 48 h at 37 °C in 24-well culture plates. Separately, the cells were incubated with arsenite at 10 µM for 24 h after pretreatment with the adenylate cyclase inhibitor SQ22536 (R & D Systems, Minneapolis, MN, USA) at 10 or 20 µM for 3 h. After treatment, the cell layer was washed twice with cold D-PBS, and 300 µL cold ISOGENⅡ reagent (Fujifilm Wako Pure Chemical Co., Ltd.) were added to each well. Cells were homogenized by pipetting. Next, 120 µL UltraPure^TM^ DNase/RNase-free distilled water (Thermo Fisher Scientific) were added to the collected samples and incubated for 15 min. The samples were centrifuged for 15 min at 12,000× *g,* and then 300 µL of the supernatant were mixed with an equal volume of 2-propanol in a separate tube. After incubation for 10 min, the samples were centrifuged for 10 min at 12,000× *g*. The supernatant was discarded by decantation, and the RNA pellet was washed twice with 75% ethanol. Finally, the RNA pellet was dissolved in RNase-free distilled water. The RNA quality and concentration were assessed by spectrophotometric analysis using a NanoDrop Lite spectrophotometer (Thermo Fisher Scientific). 

### 4.7. Reverse Transcription (RT) and Real-Time RT-qPCR

RT and real-time RT-qPCR analysis were performed as described previously [59]. cDNA synthesis was performed using 500 ng of total RNA, a ReverTra Ace^®^ qPCR RT Master Mix kit (Toyobo, Osaka, Japan), and a GeneAmp PCR system 9700 (Thermo Fisher Scientific). Real-time qPCR was performed using a THUNDERBIRD SYBR qPCR Mix (Toyobo) with 0.5 µM primers and a LightCycler 96 (Roche, Tokyo, Japan). The thermal treatment was 95 °C for 10 min, 45 cycles of 95 °C for 10 s, and 60 °C for 30 s. The primers (Table 1) were purchased from Eurofins Genomics (Tokyo, Japan). The fold change for each gene was assessed after normalization of the intensity value to that of glyceraldehyde-3-phosphate dehydrogenase (*GAPDH*).

### 4.8. Cellular ROS Assay

Endothelial EA.hy926 cells were transferred to 96-well culture plates and cultured until confluent. The cells were incubated with 2′,7′-dichlorodihydrofluorescein diacetate (H_2_DCFDA, Thermo Fisher Scientific) at 100 µM for 1 h. After treatment, the medium was discarded and the cells were washed with DMEM without phenol red. The cells were treated with arsenite at 5 or 10 µM for 0.5, 1, or 3 h. At each time point, the fluorescence intensity was measured by a Varioskan Flash multimode microplate reader (Thermo Fisher Scientific) to detect ROS production.

### 4.9. Western Blotting 

Western blotting was performed as described previously [33]. Endothelial EA.hy926 cells were seeded in 6-well culture plates and cultured until confluent in the presence or absence of siRNAs in 10% FBS-DMEM. The medium was discarded, and the cells were treated with arsenite at 10 µM for 6, 12, or 24 h in fresh 10% FBS-DMEM. After treatment, the cell layer was washed twice with cold D-PBS and collected using a hypotonic buffer [10 mM 4-(2-hydroxyethyl)-1-piperazineethanesulfonic acid (HEPES)-KOH (pH 7.9), 10 mM KCl, 1.5 mM MgCl_2_, 1 mM dithiothreitol (DTT)] and nuclear lysis buffer (20 mM HEPES-KOH (pH 7.9), 400 mM NaCl, 1.5 mM MgCl_2_, 1 mM DTT), 0.2 mM PMSF, 5% glycerol) containing protease inhibitors (cOmplete™ ULTRA Tablets, Mini, Roche). Protein concentration was determined using a detergent compatible (DC) protein assay kit (Bio-Rad, Hercules, CA, USA). Protein samples were separated by SDS-polyacrylamide gel electrophoresis and transferred to an Immobilon-P membrane (Merck KGaA). The membrane was incubated with primary antibodies against NRF2 (Novus Biologicals, Littleton, CO, USA) and Lamin A/C (Cell Signaling Technology, Danvers, MA, USA) and horseradish peroxidase (HRP)-coupled anti-rabbit IgG (Thermo Fisher Scientific) and anti-mouse IgG (GE Healthcare Japan, Tokyo, Japan) secondary antibodies. Immunoreactive bands were visualized by enhanced chemiluminescence using Immobilon Western Chemiluminescent HRP substrate (Merck KGaA) and detected with a LuminoGraph I Imaging System (ATTO, Tokyo, Japan). The band intensities were analyzed using ImageJ 1.53 g (US National Institutes of Health, Bethesda, MD, USA).

### 4.10. Statistical Analysis

All statistical analyses were performed in Excel (Microsoft, Redmond, WA, USA) with the Statcel3 add-in (OMS, Tokyo, Japan). The data were expressed as the mean ± standard deviation (S.D.). The statistical significance of data was determined using one-way analysis of variance (ANOVA) with the post hoc Bonferroni/Dunn multiple test or Student’s *t*-test as appropriate. Differences between groups were considered significant at *p* < 0.05.

## Figures and Tables

**Table 1:** Human gene-specific primers for quantitative real-time PCR.

Gene Name (Protein Name)	Forward Primer (5′–3′)	Reverse Primer (5′–3′)	Product Size (bp)
*GAPDH* (GAPDH)	GCACCGTCAAGGCTGAGAAC	TGGTGAAGACGCCAGTGGA	138
*NFE2L2* (NRF2)	GGTTCCAAGTCCAGAAGCCA	GGTTGGGGTCTTCTGTGGAG	158
*NQO1* (NQO1)	TCGTCTGTATTCCCACTTCCTTC	AGCATCTACTTCATCAGCACCATC	109
*PLAT* (t-PA)	AGCGAGCCAAGGTGTTTCAA	CTTCCCAGCAAATCCTTCGGG	93
*PLAU* (u-PA)	CCAAAATGCTGTGTGCTGCT	CCCCAGCTCACAATTCCAGT	121
*SERPINE1* (PAI-1)	CTGGCCCTTGTCTTTGGTGA	GGGTGAGAAAACCACGTTGC	138

## Data Availability

Not applicable.

## Supplementary Materials

Supplementary materials can be found at https://www.mdpi.com/1422-0067/22/2/739/s1, Figure S1: Cell viability of endothelial EA.hy926 cells after exposure to arsenite, Figure S2: Effects of arsenite on the mRNA expression of u-PA in endothelial EA.hy926 cells, Figure S3: Possible involvement of the cyclic AMP pathway in the inhibition of t-PA mRNA expression by arsenite in endothelial EA.hy926 cells.

## Abbreviations

AMP	adenosine monophosphate
ANOVA	analysis of variance
DMEM	Dulbecco’s modified Eagle’s medium
D-PBS	Dulbecco’s phosphate-buffered saline
FBS	fetal bovine serum
GAPDH	glyceraldehyde-3-phosphate dehydrogenase
H_2_DCFDA	2′,7′-dichlorodihydrofluorescein diacetate
HEPES	4-(2-hydroxyethyl)-1-piperazineethanesulfonic acid
HMECs	human microvascular endothelial cells
HRP	horseradish peroxidase
HUVECs	human umbilical vein endothelial cells
MTT	3-(4,5-dimethyl-2-thiazolyl)-2,5-diphenyl-2H-tetrazolium bromide
Nox	NADPH oxidase
NQO1	NAD(P)H quinone dehydrogenase 1
NRF2	nuclear factor E2 related factor 2
P38 MAPK	p38 mitogen-activated protein kinase
PAI-1	plasminogen activator inhibitor-1
PGI_2_	prostacyclin
ROS	reactive oxygen species
RT	reverse transcription
S.D.	standard deviation
SDS	sodium dodecyl sulfate
siRNA	small interfering RNA
t-PA	tissue-type plasminogen activator
u-PA	urokinase-type plasminogen activator
