# Supplementary material for: Arsenite Inhibits Tissue-Type Plasminogen Activator Synthesis through NRF2 Activation in Cultured Human Vascular Endothelial EA.hy926 Cells
Source: Int J Mol Sci. 2021 Jan 13;22(2):739. doi: 10.3390/ijms22020739 (PMC7828481; doi:10.3390/ijms22020739)
Supplement: Supplementary file 1 [file ijms-22-00739-s001.pdf]

Arsenite inhibits tissue-type plasminogen activator synthesis through NRF2 activation in cultured human vascular endothelial EA.hy926 cells

Tsuyoshi Nakano, Tsutomu Takahashi, Chika Yamamoto, Eiko Yoshida, Toshiyuki Kaji and Yasuyuki Fujiwara

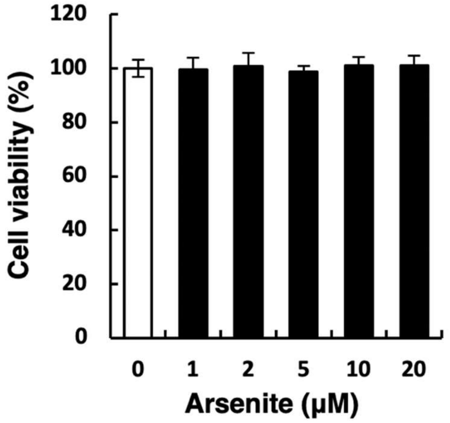

Figure S1. Cell viability of endothelial EA.hy926 cells after exposure to arsenite. Confluent cultures of endothelial EA.hy926 cells were incubated for 48 h with arsenite at 1, 2, 5, 10, or 20 μM. The data are reported as the mean ± S.D. of four samples. The data were analyzed using one-way ANOVA, followed by the Bonferroni/Dunn test.

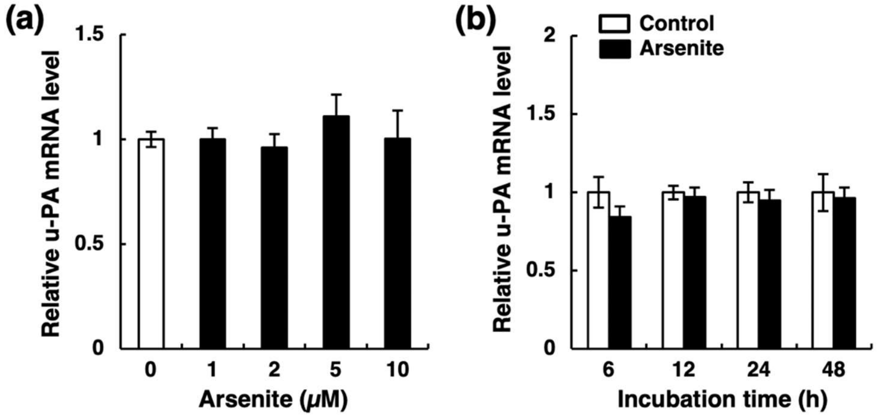

Figure S2. Effects of arsenite on the mRNA expression of u-PA in endothelial EA.hy926 cells. (a) The cells were incubated with arsenite at 1, 2, 5, or 10 μM for 24 h. The data are reported as the mean ± S.D. of three samples. The data were analyzed using one-way ANOVA, followed by the Bonferroni/Dunn test. (b) The cells were incubated with arsenite at 10 μM for 6, 12, 24, or 48 h. The data are reported as the mean ± S.D. of three samples. The data were analyzed using Student's *t*-test.

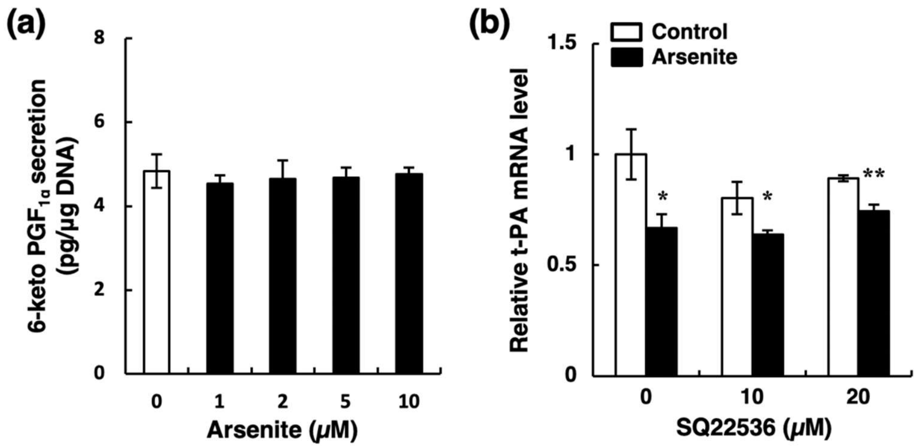

Figure S3. Possible involvement of the cyclic AMP pathway in the inhibition of t-PA mRNA expression by arsenite in endothelial EA.hy926 cells. (a) The release of PGI_2 from endothelial EA.hy926 cells into conditioned medium. The cells were incubated with arsenite at 1, 2, 5, or 10 μM for 24 h, and secreted PGI_2 was detected as 6-keto $PGF_{1\alpha}$. The data are reported as the mean ± S.D. of four samples. The data were analyzed using one-way ANOVA, followed by the Bonferroni/Dunn test. (b) Effect of SQ22536, an adenylate cyclase inhibitor, on arsenite-induced suppression of t-PA mRNA expression. The cells were incubated with arsenite at 10 μM for 24 h after pretreatment with SQ22536 for 3 h. The data are reported as the mean ± S.D. of three samples. The data were analyzed using Student's *t*-test. Significantly different from the corresponding control, $*p < 0.05$; $**p < 0.01$.
